# Patterns and drivers of female extra-pair mating in wild Kalahari meerkats

**DOI:** 10.1093/beheco/araf016

**Published:** 2025-03-06

**Authors:** Annika Herdtle, Chris Duncan, Marta B Manser, Tim Clutton-Brock

**Affiliations:** Large Animal Research Group, Department of Zoology, University of Cambridge, Downing Street, Cambridge, CB2 3EJ, United Kingdom; Kalahari Research Centre, Kuruman River Reserve, Northern Cape, 8467, South Africa; Large Animal Research Group, Department of Zoology, University of Cambridge, Downing Street, Cambridge, CB2 3EJ, United Kingdom; Kalahari Research Centre, Kuruman River Reserve, Northern Cape, 8467, South Africa; Kalahari Research Centre, Kuruman River Reserve, Northern Cape, 8467, South Africa; Department of Evolutionary Biology and Environmental Studies, University of Zurich, Winterthurerstrasse 190, 8057, Zurich, Switzerland; Mammal Research Institute, University of Pretoria, 0028, Pretoria, South Africa; Large Animal Research Group, Department of Zoology, University of Cambridge, Downing Street, Cambridge, CB2 3EJ, United Kingdom; Kalahari Research Centre, Kuruman River Reserve, Northern Cape, 8467, South Africa; Mammal Research Institute, University of Pretoria, 0028, Pretoria, South Africa

**Keywords:** cooperative breeding, extra-pair paternity, female choice, good genes, inbreeding avoidance, indirect benefits

## Abstract

In many pair-living vertebrates, females commonly mate outside the pair bond, but when and why they do so is unclear. This behavior may stem from females seeking “good genes” or “compatible genes” from extra-pair mates superior to or less related than their partner. Variation in female ability to acquire extra-pair copulations, however, may also influence extra-pair paternity rates. We analyze 23 yr of parentage data to explore extra-pair paternity in wild Kalahari meerkats (*Suricata suricatta*), cooperative breeders where a single dominant pair monopolizes most reproduction in each group. When paired with a familiar breeding partner, females almost exclusively mate extra-pair to avoid inbreeding; however, even when paired with an unfamiliar male, extra-pair paternity still occurs. In our study of unfamiliar pairings, 14% of dominant female litters contained extra-pair paternity, with 90% of offspring sired by resident dominant males, 7% by extra-group males, and 3% by subordinate immigrant males. Results were not consistent with the compatible or good genes hypotheses: more closely related dominant pairs were not more prone to extra-group paternity; extra-group sires were not less related, heavier, or older than the resident dominant male; and offspring from extra-group matings did not demonstrate advantages over within-pair offspring. Extra-group paternity was more likely when dominant females were heavier, dominant males were lighter, more extra-group males visited, and few subordinate males resided in the group, suggesting extra-pair paternity rates are primarily driven by individual and social conditions. Whether females benefit from extra-pair paternity or simply mate with any available male remains unclear.

## Teaser

When a dominant female meerkat was paired with an unfamiliar dominant male breeding partner, 10% of her offspring were not fathered by him. However, dominant females were not “trading up” to better or less related males or having better quality offspring as a result. Rather, extra-pair paternity increased when females were in good condition and had more extra-group mates available, and when dominant males were in poor condition and had few subordinate male helpers.

## Introduction

Extra-pair paternity (EPP) is common in many pair-living vertebrates and has been reported in 90% of socially monogamous bird species (Griffith et al. 2002) and 83% of socially monogamous mammals (Lambert et al. 2018). However, the frequency of EPP varies considerably between species, populations, and individuals (Petrie and Kempenaers 1998; Griffith et al. 2002; Cohas and Allainé 2009; Lambert et al. 2018). These differences may result from variation in the benefits females gain by mating extra-pair (EP) or variation in the ability of females to acquire EPP.

By engaging in extra-pair copulations (EPCs), females can gain direct benefits (ie resources obtained directly from the male) such as nuptial gifts, additional parental care, territory access, or improvements in fecundity (eg Gray 1997; Li and Brown 2002; Rubenstein 2007; Townsend et al. 2010). However, reports of direct benefits are relatively rare, and many studies focus instead on the possibility that females mate EP to obtain indirect genetic benefits such as “good genes” or “compatible genes” that improve offspring fitness (Akçay and Roughgarden 2007). Under the “good genes hypothesis,” females are expected to seek extra-pair males with superior genes. This hypothesis predicts that EPP should increase when a female’s resident partner is poor quality, that EP sires should be phenotypically superior to the resident partner, and that extra-pair young (EPY) should show higher fitness than within-pair young (WPY) sired by the cuckolded resident male (Petrie and Kempenaers 1998; Griffith et al. 2002; Neff and Pitcher 2005). For example, EP males tend to be older and larger than resident partners in socially monogamous birds (Akçay and Roughgarden 2007). Under the “compatible genes hypothesis”, rather than seeking inherently superior genes, females are expected to seek superior combinations of genes, and one way to obtain these is to mate with less related males (Zeh and Zeh 1996; Tregenza and Wedell 2000; Neff and Pitcher 2005). This hypothesis predicts that EPP should increase when the female is paired with a more closely related resident partner, that EP sires should be less related to the female than her resident partner is, and that EPY should be more outbred and fitter than WPY maternal half-siblings (Griffith et al. 2002). For example, in Savannah sparrows (*Passerculus sandwichensis*), females paired with genetically similar males are more likely to produce EPY (Freeman-Gallant et al. 2006), and in alpine marmots (*Marmota marmota*) EPY are less inbred than WPY (Cohas, Yoccoz, et al. 2007). However, in other species like the cooperatively breeding white-browed sparrow weaver (*Plocepasser mahali*), EP sires are actually more related to the female than her social mate is (Harrison et al. 2013). Additionally, patterns suggesting female choice based on indirect genetic benefits could often have alternative explanations (Griffith et al. 2002). For instance, EP sires might be larger than resident males not necessarily because of genetic advantages selected for by the female, but because larger males can better evade or overcome the resident males’ attempts to guard their mates.

As yet, neither “good genes” nor “compatible genes” benefits have been able to explain the wide variation in EPP frequencies between or within species (Akçay and Roughgarden 2007; Forstmeier et al. 2014). Females could also be mating EP as a bet-hedging strategy in unpredictable environments to increase the genetic diversity of their offspring, which may improve the likelihood of at least one offspring having traits well suited to their unknown future environment (Watson 1991; Yasui 1998, 2001; Jennions and Petrie 2000; Yasui and Yoshimura 2018). Alternatively, if competition between males selects out inferior mates, females may benefit little from being choosy and will consequently readily mate with any unfamiliar male available when they are ready to conceive.

To understand the incidence of EPP, it is important to explore not only variation in female benefits but also variation in ecological and social constraints on female mating behavior, which remain understudied (Petrie and Kempenaers 1998; Westneat and Stewart 2003; Maldonado-Chaparro et al. 2018). A reasonable prediction is that the incidence of EPP depends on the opportunity for females to mate with EP males and that this varies with the ability of resident males to guard their mates and with the capacity of females to evade their control. For instance, within bird populations, EPP rates tend to increase as breeding density increases, presumably due to increased encounters between females and EP males (Westneat and Sherman 1997; Griffith et al. 2002). Similarly, in mandrills (*Mandrillus sphinx*) (Setchell et al. 2005) and yellow baboons (*Papio cynocephalus*) (Alberts et al. 2003) alpha males tend to lose more paternity to lower-ranking males when more male competitors reside in the group. Since mate guarding is an energetically costly activity (Komdeur 2001; Low 2006) that can involve substantial reductions in foraging (eg red deer, *Cervus elaphus*: Clutton-Brock et al. 1982; olive baboons, *Papio anubis*: Packer 1979; yellow baboons, *Papio cynocephalus:*Alberts et al. 1996; Seychelles warblers, *Acrocephalus sechellensis*: Komdeur, 2001), the ability of males to monopolize their partner’s reproduction is likely to depend on their own condition and how much energy they can dedicate towards mate guarding. Likewise, if seeking out EP males involves energetically expensive behaviors or detracts from foraging, females may also be constrained by their own body condition. It remains unclear how these potential constraints and benefits interact to influence EPP patterns within populations, particularly in pair-living mammals.

In this study, we use a multigenerational pedigree and observational data on wild meerkats (*Suricata suricatta*) collected over 30 yr to (i) investigate how variation in the incidence of EPP is related to social and individual factors, (ii) compare traits of EP sires and the resident male partner, and (iii) compare the characteristics of EPY and WPY. Meerkats are cooperative breeders living in groups of up to 50 individuals with a single dominant female and a single dominant male. Females can acquire dominance natally or in a newly formed group after being evicted from their natal group, but do not immigrate into established groups (Clutton-Brock, Brotherton, et al. 1998; Clutton-Brock and Manser 2016; Duncan et al. 2018). Males only acquire dominant breeding positions non-natally by dispersing and immigrating into an established group or forming a new group with evicted females (Clutton-Brock, Brotherton, et al. 1998; Duncan et al. 2023). The dominant pair monopolizes the majority of reproduction, with approximately 90% of pups being the offspring of dominant females and 80% being the offspring of dominant males (Griffin et al. 2003; Hodge et al. 2008).

Following the death of a previous dominant male, natal males can sometimes become behaviorally dominant (Clutton-Brock and Manser 2016; Spence-Jones et al. 2021). In 75% of these cases, the natal dominant male is the offspring, brother, or half-brother of the dominant female, posing substantial inbreeding risk (Spence-Jones et al. 2021). Similarly, when a female gains dominance in her natal group, if the non-natal dominant male immigrated into her group before she was born, he is likely to be her father or uncle. Previous work has shown that dominant females rarely breed with these familiar dominant males, producing offspring with EP males instead (Nielsen et al. 2012; Duncan 2021; Spence-Jones et al. 2021). However, inbreeding is still a risk even when paired with an unfamiliar immigrant male, with cases of moderate inbreeding (inbreeding coefficients between 0.125 and 0.25) between unfamiliar individuals being documented and having negative effects on meerkat survival and morphometric traits (Nielsen et al. 2012). If dominant females paired with unfamiliar males are able to assess their relatedness to mates using strategies like phenotype-matching, they might use EP mating to avoid inbreeding with unfamiliar dominant male relatives and instead seek out less related EP males.

When paired with an unfamiliar dominant male, between 8% and 17% of dominant females’ offspring are EPY, being sired either by extra-group (EG) males or intra-group (IG) immigrant subordinate males (Griffin et al. 2003; Spong et al. 2008; Leclaire, Nielsen, Sharp, et al. 2013; Duncan 2021). EP males can sire entire litters or share paternity with the dominant male or other EP males in mixed-paternity litters (Griffin et al. 2003). Leclaire, Nielsen, Sharp, et al. (2013) found that when dominant females were paired with a more closely related unfamiliar immigrant dominant male, a higher proportion of the pair’s total offspring were EPY, suggesting females may mate EP to seek more compatible genes. However, the same study showed that EP sires were no less related to the dominant females than the resident dominant males were, and EPY were no more outbred or heterozygous than WPY, casting doubt on the idea that compatible genes benefits drive EPP. Spong et al. (2008) found that social dynamics influenced EPP, with higher rates observed in groups with more subordinate males and frequent visits from extra-group males. The role of female traits and potential “good genes” benefits, however, remain less clear. In our study, we build on previous research by incorporating over eight additional years of data to examine the conditions under which dominant females mate EP despite having an unfamiliar dominant male partner available. We evaluate both “compatible genes” and “good genes” as potential drivers of female EP mating, while also considering how individual condition and social factors can influence mate availability and mate guarding. Specifically, we explore how traits of the dominant male and female, their relatedness, and their social environment predict the likelihood of the dominant female’s litter containing EPP. We then test whether the EP sires are less related, older, or heavier than the resident dominant male. Finally, we compare survival, weight, and dominance acquisition between EPY and WPY.

## Methods

### Study system

This study used data on a wild population of meerkats at the Kalahari Research Centre in the Kuruman River Reserve and neighboring ranchland in the Northern Cape of South Africa (26°59’S, 21°50’E). Data was collected between 1993 and August 2023, incorporating 4,201 individuals living in 164 social groups. One to two individuals per group, usually the dominants, were fitted with VHF collars, most individuals were tagged with subcutaneous RFID transponder chips, and all individuals were marked with unique dye marks to allow for identification. The study animals were habituated to close observation (<1 m), with most individuals also trained to step onto an electronic scale in exchange for a small reward of egg crumbs or water.

Groups were visited approximately once every 3 d, when group compositions, births, deaths, EG visitors, weights, and dominance behaviors were recorded. Births were determined by sudden drops in female weight, while dominants could be easily distinguished from subordinates based on the direction and frequency of aggressions and submissions and the higher frequency of anal marking (Clutton-Brock, Gaynor, et al. 1998; Kutsukake and Clutton-Brock 2006, 2008; Madden et al. 2011). Research protocols followed guidelines from the Association for the Study of Animal Behavior and were approved by the University of Pretoria Ethics Committee (EC047-16, EC010-13, SOP029-12) and the Northern Cape Province Department of Environment and Nature Conservation, South Africa (FAUNA 1020/2016).

### Genetic analysis

DNA was extracted from tail tip samples of pups born within the study population and individuals who immigrated in. Samples from 1993 through August 2019 were genotyped at up to 18 polymorphic microsatellite loci, following protocols by Nielsen et al. (2012). Using the microsatellite data and lists of candidate parents, the program COLONY2 (Wang 2004) was able to infer parentage and sibships. Candidate mother lists consisted of all females who were pregnant and gave birth in the pup’s natal group around the recorded birth date, while candidate father lists consisted of all genotyped males alive in the study population within 2 wk of the estimated conception date. Only parentage assignments with a confidence over 80% were retained. Since some individuals had their parentage assessed more than once as new genetic data became available, if there were conflicting assignments, the most recent one was used. If a genetic mother could not be assigned and only one pregnant female was observed in the field, we assigned this female as the mother. Using the parentage assignments and the *ggroups* package in R (Nilforooshan and Saavedra-Jiménez 2020), we constructed a 12-generation pedigree from which we calculated relatedness coefficients.

### The pup dataset

Between October 1993 and August 2019, 1,595 pups that were conceived by a dominant female who was paired with a dominant male emerged from their birth burrows and were genotyped at 9 or more loci (mean = 17.5 loci). This constituted 441 litters from 146 dominant pairs. Conception was estimated at 70 d prior to birth, with a conception window from 63 to 77 d prior to birth (Doolan and Macdonald 1997; Young et al. 2007; Spong et al. 2008; Leclaire, Nielsen, Sharp, et al. 2013). Prior studies have shown that natal dominant males and dominant males who immigrated into the dominant female’s natal group before she was born rarely breed with the dominant female because they are usually familiar close relatives (Nielsen et al. 2012; Clutton-Brock and Manser 2016; Duncan 2021; Spence-Jones et al. 2021). Our study aimed to investigate why dominant females are still documented mating extra-pair even when paired with an unfamiliar dominant male. Therefore, we excluded 186 pups (57 litters; 24 pairs) that were conceived when the dominant female was paired with a natal dominant male and 80 pups (22 litters; 10 pairs) conceived when the dominant female was paired with a dominant male who immigrated into her group before her birth. We additionally excluded cases where the dominant male was present in the group upon the group’s initial discovery and habituation because we could not know whether the male was natal or immigrant (32 pups; 11 litters; 5 pairs). The remaining dataset consisted of 1,297 pups in 351 litters from 106 unfamiliar dominant pairs.

We then sought to determine whether each pup’s father was the resident dominant male, an IG subordinate male, or an EG male. COLONY2 was able to assign a population male as the father with over 80% confidence for 965 out of 1,297 pups (mean confidence of accepted assignments = 99.8%, with 97% of assignments having 100% confidence). Father identities for an additional 102 pups were able to be assigned by re-analysis of sibships identified by COLONY2, examination of inferred “dummy” parent genotypes, and comparison with MASTERBAYES (Hadfield et al. 2006) assignments (see Nielsen et al. 2012). For the remaining 230 pups, no identified population male was assigned as father. Eliminating all these pups with unresolved paternity would generate bias against EG paternity, since some EG sires are likely to be unsampled males residing outside the study population. Instead, we constrained the dataset to pups born into groups where all IG candidate fathers (ie the dominant male and every subordinate immigrant male resident during the conception window) were genotyped at 9 or more loci (82%, 1059/1297 of pups).

With all the IG candidate fathers genotyped, unresolved paternities (125 pups) potentially represent cases where the pup was sired by an unsampled EG male. However, it is also possible that COLONY2 could not distinguish between two highly related candidate fathers within the study population (IG or EG) and thus was unable to confidently assign a single paternity. In order to retain only the instances where the true father was an unsampled EG male, we used CERVUS 3.0.7 (Kalinowski et al. 2007) to conduct an elimination analysis, testing whether we could exclude all IG candidate fathers with a confidence of at least 95%. Following Lemons et al. (2015), we conducted a simulation of paternity analysis (simulation parameters described in supplementary materials) to determine a critical logarithm of odds (LOD) score that provides 95% confidence that none of the IG candidate fathers are the true father. For each of the pups with unresolved paternity, we then performed paternity analysis, supplying only the IG candidate males as candidate fathers. This analysis provided an LOD score for each offspring / candidate father pair, indicating the likelihood of that male being the offspring’s true father. For 48 of the 125 pups with unresolved paternity, all IG candidate fathers had an LOD score below the critical 95% LOD threshold determined by simulations, meaning that we could confidently eliminate all within-group males as the potential father and infer that the pup was sired by an EG male. For the remaining 77 pups, at least one of the IG candidate fathers had an LOD score above the critical LOD threshold, meaning that we could not confidently exclude them as the true father, and we instead eliminated these pups from our dataset. This elimination should not bias the paternity-type estimates, as these unresolved paternities most likely reflect scenarios where multiple compatible candidate fathers existed within the group, or where at least one compatible candidate was present both within and outside the group, preventing COLONY2 from confidently assigning paternity to a single male. In such cases, the unknown true father could still be an EG male, an IG subordinate, or the IG dominant. The final dataset consisted of 982 pups in 264 litters from 80 dominant pairs. Additional individual and group composition metrics were calculated as described in Table 1.

**Table 1.** Descriptions of variables included in the EG-EPP model.

Term	Mean (Range) or Categories	SD	Description
**Dominant female** **natal status**	Natal *(120 litters; 24 females)* Non-natal *(109 litters; 25 females)*		Did the female acquire dominance and breed in her natal group or in a new non-natal group after being evicted from her natal group?
**Pair relatedness:** ^1^	0.091 (0 to 0.408)	0.110	Pedigree relatedness between the dominant male and dominant female. If both parents of one or both dominants were unknown, relatedness was assumed to be 0.
**Dominant male weight**	760 (583 to 947)	69	Mean dominant male pre-foraging morning weight over a 4-wk window^2^ surrounding conception (g).
**Dominant female weight**	772 (570 to 943)	70	Mean dominant female pre-foraging morning weight over a 4-wk window ^2^ surrounding conception, excluding days when pregnant with a previous litter (g).
**EG rovers**	0.11 (0 to 1.11)	0.21	Mean number of distinct identifiable EG males seen visiting the group per observation day during the 2-wk conception window.
**Adult subordinate males**	3.8 (0 to 13.3)	2.9	Mean number of adult (≥1 yr old) subordinate males residing in the group during the 2-wk conception window.
**Dominant male** **tenure length**	564 (8 to 1,829)	431	Days between the start of the dominant male’s tenure and the estimated conception date.

^1^Descriptive statistics for relatedness were calculated based on unique pairings because some pairs had more than one litter in the dataset.

^2^A 4-wk conception window (56 to 84 d prior to birth) was used to calculate mean weights instead of the 2-wk conception window (63 to 77 d prior to birth) used for other metrics in order to allow for more litters to be incorporated into the analysis since several dominant individuals did not have recorded weights within the 2-wk conception window.

### Statistical analysis

As the majority of EPP was found to originate from EG males (see results), we focused the statistical analysis only on extra-group extra-pair paternity (EG-EPP). We did not model EPP from EG and IG subordinate males together because different factors may influence these two EPP sources.

#### Factors affecting the probability of EG-EPP.

To investigate how individual and social factors relate to EG-EPP, we modeled the probability of a dominant female litter containing EG-EPP using a Bayesian logistic mixed-effects model with a Bernoulli distribution. We grouped pups into litters and set the binary response variable to one when the litter contained at least one pup sired by an EG male, and zero when it did not. The model included random effects for dominant female and dominant pair identities. Fixed effects included dominant female natal status, pedigree relatedness between the dominant pair, dominant male weight, dominant female weight, the frequency of visits from EG male rovers, the number of adult subordinate males in the group, and dominant male tenure length (Table 1). Rovers are prospecting males that make extraterritorial forays in search of reproductive or dispersal opportunities and are typically met with aggression from resident males in the groups they approach (Doolan and Macdonald 1996; Mares et al. 2012; Mares et al. 2014). We included female natal status since meerkats may be limited in their ability to precisely determine genetic relatedness to potential mates and may instead rely on proxies such as their own natal status. It is possible that litter size could be a confounding factor in EPP analyses, as we may be more likely to detect EPP in litters with a greater number of genotyped pups. However, this did not appear to be the case in our study, where litter size had no clear effect on the likelihood of EG-EPP (Estimate [95% CI] = −0.41 [−2.12 to 1.24]). We therefore did not include litter size as a predictor in the final model to avoid overcomplication.

We tested quadratic terms for pair relatedness and subordinate male numbers, as we had a priori reasons to suggest these effects may not be linear. To assess the impact of these quadratic terms on model predictive performance, we conducted repeated k-fold cross-validation with k = 10 and five repeats, then averaged pointwise expected log predictive density (elpd) estimates for each model across these repeats. For quadratic terms retained in the final model, we conducted post-hoc analyses to investigate the non-linear effects in greater detail. Specifically, we split the dataset at the value of the predictor where the model estimated the likelihood of EG-EPP to be at its minimum or maximum (ie the vertex of the quadratic curve). This created two subsets of data, which we analyzed separately to test for significant effects below and above the vertex. Due to decreased sample size in these subsets, models were simplified when necessary by including only the strongest and most certain predictors to ensure model convergence.

Data for the EG-EPP model included 229 litters from 72 dominant pairs, since information on some predictors was not available for all 264 litters. This analyzed dataset represents 65% of all litters (229/351) born to dominant females paired with an unfamiliar dominant male. When one or both dominants had no identifiable parents, the dominant pair was assumed to be unrelated. To check whether this assumption affected the estimated relationship between pair relatedness and EG-EPP, we fitted a conservative second model using a subset of data restricted to pairs where both dominants had two parents of known identity (101 litters, 31 pairs). The results for pair relatedness were consistent between the main model and the conservative model and are reported in the supplementary materials (Table S2).

#### Comparing EG sires to the resident dominant male.

To test whether females exhibited a mating a preference for less related or higher quality males, we compared the relatedness, ages, and weights of identifiable EG sires to those of the cuckolded dominant male. If females mate EP to secure good genes, we predicted that EP sires would be larger and older than the resident dominant male. Increased body weight could signal advantageous genetic traits, such as superior foraging ability or health, which, if heritable, can benefit meerkat offspring by enhancing their survival chances and success in dominance contests (Clutton-Brock et al., 2001; Hodge et al., 2008; English et al., 2013; Duncan et al., 2018; Duncan, 2021). Similarly, older males may be preferred because their age reflects their ability to survive, which could suggest good quality genes (Kokko and Lindström, 1997).

We utilized two-sided paired t-tests, or Wilcoxon signed-rank tests when values were not distributed normally, to compare the EG sire and dominant male. We compared the males’ relatedness to the dominant female using a dataset comprised of ten unique combinations of the dominant female, dominant male, and EG sire. We then conducted a conservative follow-up analysis restricted to the six cases where the dominant male, dominant female, and EG sire all had both parents identified, ensuring a more reliable pedigree. The results of this conservative analysis were consistent with those of the main comparison and are reported in the supplementary materials. We compared the males’ ages at conception using a dataset containing nine unique combinations of dominant males and EG sires, where both males were born within the study population and had known birthdates. We compared the mean weights of eight unique combinations of dominant males and EG sires, with weights being averaged over a 4-wk window around conception (56 to 84 d before birth) and each male having at least two weights (mean = 9.6 weights) during this period. There were no instances in these datasets where the dominant male was cuckolded by the same EG sire multiple times at different ages or weights. Overall, sample sizes were limited as most EG paternity (71%) originated from unidentified males outside the study population, for whom we do not have pedigree, age, or weight data.

#### Comparing EG-EPY and WPY.

To determine how paternity type affects offspring, we compared survival, weight, and dominance acquisition between extra-group extra-pair young (EG-EPY) and WPY maternal half-siblings using Bayesian mixed-effects models. For each analysis, we only included dominant pairings that had produced both EG-EPY and WPY which could be incorporated into the given dataset.

We conducted two models of survival, one assessing survival from emergence to nutritional independence (90 d) (n = 250 pups), and another assessing survival from nutritional independence to adulthood (365 d) (n = 217 pups). These models used Bernoulli distributions, where the response variable was set to one if the offspring survived until the given benchmark and zero if it did not. Random effects included dominant pair identity and year, while fixed effects included father type (dominant or EG), mean number of pups (individuals < 90 d old) in the group, mean number of helpers (individuals > 180 d old) in the group, and birth month. For the emergence-to-independence model, we averaged the numbers of helpers and pups from the offspring’s birth until either the offspring’s death (if the offspring did not survive to independence) or 90 d after birth (if the offspring did survive to independence). For the independence-to-adulthood model, we averaged the numbers of helpers and pups from 90 d after birth until either the offspring’s death (if the offspring did not survive to adulthood) or 365 d after birth (if the offspring did survive to adulthood). We fitted birth month as a cyclic cubic spline and tested for a quadratic effect of helper number. We also included offspring sex as a fixed effect in the independence-to-adulthood model but not the emergence-to-independence model, since some of the offspring that died very young had not yet been sexed.

We conducted two models of offspring weights, one assessing weight at nutritional independence (n = 189 pups) and one assessing weight at adulthood (n = 154 pups), both using a Gaussian distribution. Pre-foraging morning weights were averaged over a 2-wk window (83 to 97 and 358 to 372 d after birth, respectively). We included random effects for dominant pair identity and year, and fixed effects for father type, offspring sex, average age of the offspring on the day weighed, mean number of pups in the group, mean number of helpers in the group, rainfall, and birth month fitted as a cyclic cubic spline. Both helper and pup counts were averaged over the 60 d leading up to to the given benchmark. Rainfall was calculated as a cumulative total from 60 to 2 d before each benchmark, based on previous research linking rainfall in the previous 2 mo to meerkat mass and growth (English et al. 2014). We used daily rainfall data from the Global Precipitation Climatology Project provided by the NOAA PSL (Boulder, Colorado, USA, https://psl.noaa.gov/) (Adler et al. 2003). To account for potential non-linear effects of rainfall and helper number, we tested models with different combinations of these quadratic terms and compared predictive performance using leave-one-out cross-validation.

To compare the probability of EG-EPY and WPY acquiring dominant breeding positions within the study population, we used a model with a Bernoulli distribution, where the response variable was set to one when the offspring acquired and held dominance for at least one 30-d period during their observed lifetime and zero when they did not (n = 166 pups). For male offspring, we only considered dominance positions acquired non-natally, since holding a natal dominance position does not confer reproductive benefits to males (Spence-Jones et al. 2021). We included dominant pair identity as a random effect and included father type and offspring sex as fixed effects. Analysis was restricted to offspring that had survived at least 1 yr, as dominance is rarely acquired before this age. Available dominance data spans until August 2023, and target offspring who were still alive at this time were incorporated in the analysis since all were at least 4 yr of age by this date, and 92% of all dominants began their first dominance bout before the age of four.

All data processing and analysis was performed using R Statistical Software (v4.2.2; R Core Team 2022). Bayesian mixed-effects models were fitted using the R package *brms* (Bürkner 2018). Multicollinearity was examined using correlation matrices and variance inflation factors (VIF), with values over 2 considered to indicate problematic collinearity (Zuur et al. 2010). All continuous predictor variables were centered and scaled by two standard deviations (2SD) prior to modeling (Gelman 2008). The uncertainty of coefficient estimates was assessed using 95% credible intervals drawn from posterior distributions of the models. For models with Gaussian distributions, all coefficient estimates and credible intervals for continuous predictors are reported as the change in response variable units associated with a 2SD increase in the predictor. For models with Bernoulli distributions, estimates represent the change in log-odds associated with a 2SD increase in the predictor. For categorical predictors, estimates reflect the change in response variable units (Gaussian models) or log-odds (Bernoulli models) when the predictor is set to the specified category compared to the reference category.

## Results

### Frequency of EPP

**Fig. 1.**
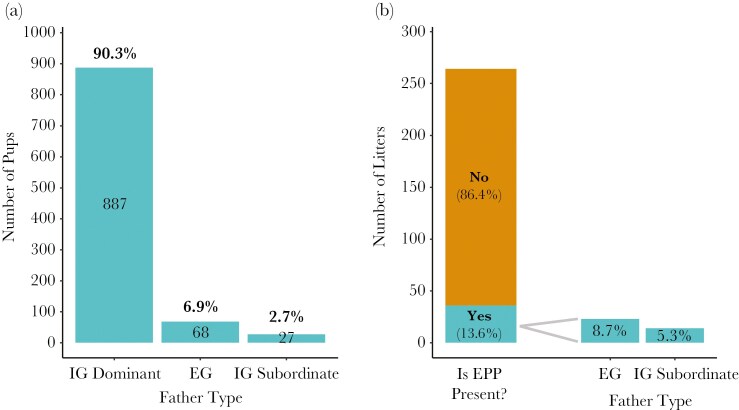
Paternity of dominant females’ a) pups and b) litters, with EPP divided into paternity from extragroup (EG) males and intra-group (IG) subordinate males. Litters with EPP presence contained at least one emergent genotyped pup sired by an extra-pair male.

This study examined data from 982 pups in 264 litters born over 22.5 yr during 80 pairings between 53 dominant females and 73 unfamiliar dominant males in 32 different groups. Dominant males sired 90.3% (887 pups) of the dominant females’ pups, with 9.7% (95 pups) being sired by EP males. More specifically, 6.9% (68 pups) were sired by EG males and 2.7% (27 pups) by IG subordinate males (Fig. 1a). At the litter level, 86.4% (228 litters) were sired exclusively by the dominant male, while 13.6% (36 litters) included at least one EP pup (Fig. 1b). 8.7% (23 litters) of litters contained EG paternity and 5.3% (14 litters) contained IG subordinate paternity; one litter contained both EG and IG subordinate paternity. Although EG-EPP was more common than EPP from IG subordinate males (IG-EPP) overall, IG-EPP was very unlikely to occur in many litters due to the absence of subordinate immigrant males in the group during the conception window for 60% of litters (159/264). In groups where at least one subordinate immigrant male resided at some point during the conception window, 6.9% of the dominant females’ pups (26/378) and 12.4% of her litters (13/105) contained IG subordinate paternity.

Though only 9.7% of pups were EP overall, 38% of dominant females (20/53) and 31% of dominant pairings (25/80) had at least one litter containing EPP, indicating that EPP was not isolated to only a small subset of females or pairs.

Among the 248 litters that contained more than one genotyped emergent pup, 220 were sired completely by a single male, and 17 were sired by two males. In an additional 9 litters, all pups were sired by unsampled EG males, so the exact number of different fathers is unclear. In the remaining 2 litters, at least two fathers were involved since at least 1 pup was sired by the dominant male, and at least two pups were sired by one or more unsampled EG males. When EP males sired offspring in a dominant female’s litter, EG males were less likely to share paternity with the dominant male (26%, 6/23 litters) than IG subordinate males were (57% 8/14 litters), a trend noted by Griffin et al. (2003).

### Extra-pair fathers

The 27 pups sired by IG subordinate fathers were the offspring of 15 unique males, all but one of which were immigrants to the dominant female’s group. Most of these immigrants (13/14 sires) were known to have originated from the same natal group as the dominant male and immigrated on the same day (10/14 sires) or within 9 d (2/14 sires) of him. Four were full siblings of the dominant male, six were at least maternal half-siblings, and one was the father of the dominant male. Parentage analysis indicated that one pup was the product of a dominant female mating with her 9-mo-old natal son, therefore cuckolding his own father, although this would seem unlikely given the male’s age and the rarity of parent-offspring inbreeding.

Most pups with EG paternity (48/68) were the inferred offspring of unidentified EG fathers, but 20 pups were sired by 10 different identifiable EG fathers within the study population. Of these 10 EG sires, 7 were related to the cuckolded dominant male by a relatedness coefficient of at least 0.25, including two full siblings, two half-siblings, and two nephews.

### Do females mate EG to breed with less related males?

**Fig. 2.**
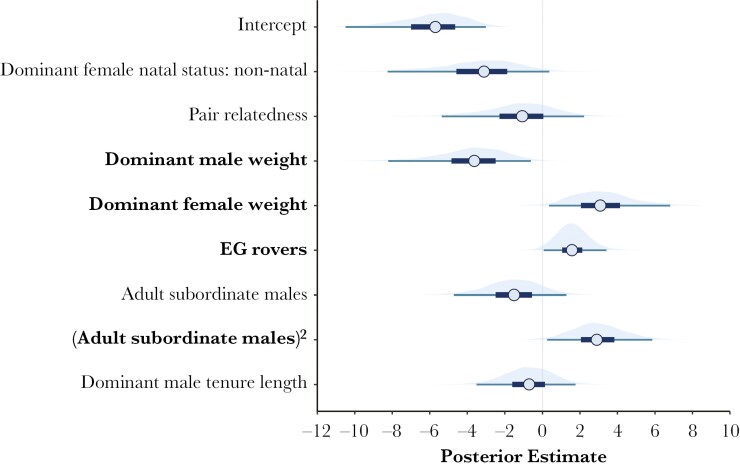
Effect size plot for the EG-EPP model, showing the effect of predictors on the probability of dominant female litters containing EG-EPP. The plot depicts posterior means (points), 50% credible intervals (thick lines), 95% credible intervals (thin lines), and the distributions of posterior draws (density plots) for each fixed effect in the model. The Bayesian mixed-effects model used a Bernoulli distribution and included random effects for dominant female and dominant pair identities. All continuous predictor variables were centered and scaled by 2 SD before modeling. Estimates are reported on the logit link scale, representing a change in the log-odds of EG paternity in response to a 2SD increase in the continuous predictor or a change in “dominant female natal status” from the reference category of “natal” to the category “non-natal.” The model included 229 litters from 72 dominant pairings. Predictors whose 95% credible intervals did not cross zero are bolded.

**Fig. 3.**
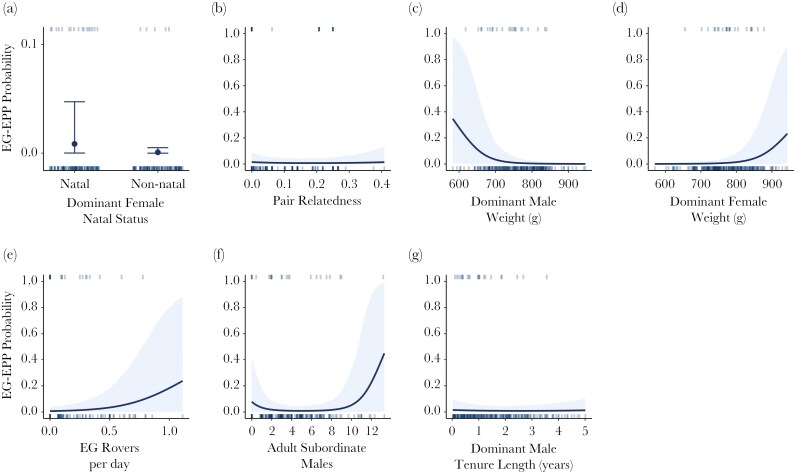
Effects of social and individual factors on the probability of at least one pup in a dominant female’s litter being sired by an EG male. The plots depict predicted mean probability estimates (lines / points) and 95% credible intervals (shaded regions / error bars) for the full range of predictor values (vertical ticks). Predictions were calculated while holding all other continuous predictors constant at their means and setting dominant female natal status to “natal.”

**Fig. 4.**
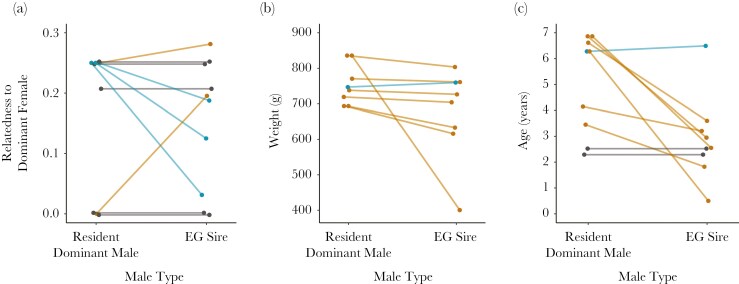
Paired comparisons of the EG sire’s and the resident dominant male’s a) relatedness to the dominant female (n = 10), b) weight (n = 8), and c) age (n = 9) at conception. Blue lines signify that the direction of difference matched predictions from the “compatible genes” or “good genes” hypotheses, orange lines signify the direction of difference was opposite of predictions, and gray lines signify no difference in value. In Plot A, the gray lines and points at 0 and 0.25 have been jittered vertically to clearly display that two sets of males were compared at each of these relatedness values.

The pedigree relatedness between the dominant female and dominant male did not affect the likelihood of their litter containing EG-EPP (Fig. 2, Fig. 3b, Table S1). Adding a quadratic term for relatedness did not reveal any quadratic effect (Estimate [95% CI] = −0.21 [−9.73 to 8.25]) and did not improve the model’s predictive performance (Δelpd ± SE = −1.7 ± 0.4), so it was not included in the final model. EG sires did not differ significantly from the resident dominant male in terms of pedigree relatedness to the dominant female (n = 10, paired t-test: *t*_*9*_ = 0.530, *P* = 0.609; EG sire mean = 0.153, dominant male mean = 0.171, mean paired difference = 0.018) (Fig. 4a).

Even though we excluded cases where natal dominant females were paired with familiar dominant males who were likely to be close relatives (ie natal males or males who immigrated into her group before her birth), there was still a trend towards natal dominant females being more likely to have litters containing EG-EPP (12.9%, 17/132 litters) than dominant females breeding outside their natal group (4.7%, 6/128 litters) (Fig. 2, Fig. 3a, Table S1). There was, however, no difference in dominant pair relatedness between females breeding natally versus non-natally (Mann-Whitney U: *W* = 673.5, *P* = 0.394; natal mean_n = 42 pairs_ = 0.091 and non-natal mean_n = 36 pairs_ = 0.080).

### Do females mate EG to breed with phenotypically “superior” males?

Though dominant females were more likely to acquire EG-EPP when paired with lighter dominant males (Fig. 2, Fig. 3c, Table S1), EG sires were still significantly lighter than the resident dominant males they cuckolded (n = 8, Wilcoxon signed-rank: *W* = 33, *P* = 0.039) by a median 15 g (mean = 28 g, sd = 31 g) (Fig. 4b). EG sires were also significantly younger than the resident dominant males (n = 9, paired t-test: *t*_*8*_ = 2.952, *P* = 0.018), by a median 1.6 yr (mean = 2.2 yr, sd = 2.2 yr) (Fig. 4c). When excluding one outlier EG sire who was apparently only 6 mo old at conception and therefore very light, EG sires were still significantly younger (paired t-test: *t*_*7*_ = 2.624, *P* = 0.034), but the difference in weight was no longer significant (paired t-test: *t*_*6*_ = 2.328, *P* = 0.059).

### Do offspring from EG matings have advantages over WPY?

**Fig. 5.**
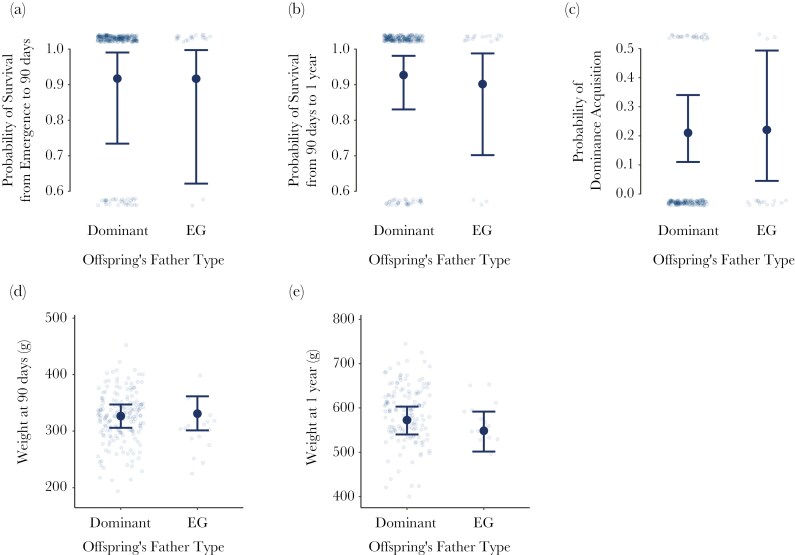
Comparisons of EG-EPY and WPY maternal half-siblings. Plots depict offspring a) survival from emergence to nutritional independence (n = 250 pups), b) survival from nutritional independence to adulthood (n = 217 pups), c) probability of acquiring a dominant breeding position within the study population (n = 166 pups), d) weight at nutritional independence (n = 189 pups), and e) weight at adulthood (n = 154 pups). Translucent points represent individual offspring, with points above and below the scale representing offspring that did and did not, respectively, survive (a, b) or acquire dominance (c). Solid blue points and bars depict the predicted mean probability estimates and 95% credible intervals, respectively, when all other continuous variables were set to their mean, pup sex was set to female, and birth month was set to January. These estimates and credible intervals originate from Bayesian mixed effects models with Bernoulli (a, b, c), or Gaussian (d, e) distributions.

Dominant females’ offspring sired by EG males did not differ in survival, weight, or dominance acquisition from their WP maternal half-siblings sired by the resident dominant male. In these models, WPY were used as the reference category, and estimates describe the difference for EG-EPY relative to WPY. Father type did not influence survival from emergence to nutritional independence (90 d old), with 90% of EG-EPY surviving (19/21 pups) and 87% of WPY surviving (199/229 pups) (Estimate [95% CI] = 0.35 [−1.81 to 2.64]) (Table S3a, Fig. 5a). Similarly, survival from independence to adulthood (365 d old) was not affected by whether the pup was sired by an EG male (84% survival; 16/19 pups) or the resident dominant male (84% survival; 167/198 pups) (Estimate [95% CI] = −0.17 [−1.68 to 1.59]) (Table S3b, Fig. 5b). Father type did not affect offspring weights at nutritional independence (mean ± sd = 301 ± 42 g for EG-EPY [n = 16 pups], 324 ± 45 g for WPY [n = 173 pups]) (Estimate [95% CI] = 4.35 [−18.11 to 26.86]) (Table S3d, Fig. 5d) or at adulthood (mean ± sd = 569 ± 45 g for EG-EPY [n = 15 pups], 587 ± 65 g for WPY [n = 139 pups]) (Estimate [95% CI] = −24.09 [−59.45 to 12.50]) (Table S3e, Fig. 5e). Finally, father type did not influence the probability of offspring acquiring a dominant breeding position within the study population, which occurred for 19% of EG-EPY (3/16 pups) and 17% of WPY (25/150 pups) (Estimate [95% CI] = −0.05 [−1.68 to 1.32]) (Table S3c, Fig. 5c).

### Is the likelihood of EPP from EG sires related to individual and social conditions?

Litters were more likely to contain EG-EPP when dominant males weighed less (Fig. 2, Fig. 3c, Table S1), when dominant females weighed more (Fig. 2, Fig. 3d, Table S1), and when a greater number of identifiable EG rovers were observed visiting the dominant pair’s group (Fig. 2, Fig. 3e, Table S1). Dominant male tenure length did not have a notable effect on the likelihood of EG-EPP (Fig. 2, Fig. 3g, Table S1).

Our analysis suggested a non-linear relationship between the number of subordinate males and the likelihood of EG-EPP, as the quadratic term had a credible interval that did not cross zero (Fig. 2, Table S1). However, k-fold cross-validation indicated that including the quadratic term did not significantly improve predictive accuracy (Δelpd ± SE = −1.1 ± 1.9 SE). Since our focus was on understanding relationships rather than predictive accuracy, we choose to include the quadratic term for subordinate males in the final model and investigate this effect further with post-hoc tests after splitting the data around the vertex of the quadratic curve (5.3 adult subordinate males). When there were less than 5.3 subordinate males residing in the group (171 litters), the likelihood of EG-EPP increased the fewer males there were (Estimate [95% credible interval] = −5.47 [−12.64 to −1.04]) (Fig. S1a). When there were at least 5.3 subordinate males in the group (58 litters), there was no clear effect of subordinate male numbers on the probability of EG-EPP (Estimate [95% credible interval] = 1.21 [−4.22 to 7.08]) (Fig. S1b). This result suggests that having more subordinate males in a group may help prevent EG-EPP; however, this effect diminishes as the number of subordinate males grows, eventually reaching a point where additional subordinates no longer impact EG-EPP rates.

## Discussion

### Evolutionary benefits of EPP

Our results provide no evidence that EP mating offers indirect genetic benefits to dominant female meerkats paired with unfamiliar dominant males. Pedigree relatedness between these dominant pairs in our study ranged from r = 0 to r = 0.408, presenting the potential risk of inbreeding, which can negatively affect meerkat traits such as mass at emergence, growth until independence, and juvenile survival (Nielsen et al. 2012). Given this risk, we expected dominant females paired with closely related unfamiliar dominant males to avoid inbreeding by seeking out less-related EP mates who could provide more compatible genes for their offspring. However, our findings showed that dominant females more closely related to their dominant male partner were no more likely to acquire EG paternity than dominant females less related to their partner, a finding that contrasts with earlier results from Leclaire, Nielsen, Sharp, et al. (2013). Furthermore, when dominant females did breed EG, the EG sires were no less related to them than their dominant male partners, a finding consistent with Leclaire, Nielsen, Sharp, et al. (2013). We also found no evidence of a preference for intermediate levels of inbreeding, as has been found in alpine marmots (Cohas et al. 2006, 2008; Ferrandiz-Rovira et al. 2016). These results suggest that dominant females may be limited in their ability to selectively choose less related mates due to the challenges of discriminating between unfamiliar kin and non-kin. While a previous experiment suggested meerkats may use odor-based kin discrimination, applications of this ability remain unclear (Leclaire, Nielsen, Thavarajah, et al. 2013).

Reliance on relatedness proxies like familiarity or natal status may not always be precise, which could cause some natal females to err on the side of caution. A female breeding non-natally after eviction faces a relatively lower risk of inbreeding since both sexes in newly formed groups originate from different natal groups. In contrast, a natal dominant female faces a higher inbreeding risk because her dominant male partner could be her natal brother or her immigrant father or uncle who joined her natal group before her birth (Spence-Jones et al. 2021). Prior research shows that natal dominant females avoid breeding with these familiar dominant males (Duncan 2021; Spence-Jones et al. 2021). However, we found that even with these familiar males excluded, there was still a trend towards natal dominant females being more likely to mate EG than non-natal dominant females, despite there being no difference in their relatedness to the dominant male. This trend may be due to natal females struggling to discriminate between natal males and males who immigrated into their group before their birth (posing an inbreeding risk) versus later-arriving immigrants (who are no more likely to be a close relative than any other EG male), having some level of familiarity with both. This lack of precision in the familiarity proxy could cause natal dominant females to seek EG mates, even when paired with an unrelated immigrant male. This trend aligns with findings in the closely related banded mongoose (*Mungos mungo*), where EG paternity correlates with proxies like female natal status rather than direct relatedness to breeding males (Wells et al. 2021). It is important to note, however, that there was uncertainty in the trend we observed, with the 95% credible interval crossing zero, suggesting more data may be needed to clarify the effect of female natal status.

Dominant females do not appear to breed with EG males to acquire good genes from phenotypically “superior” mates. EG sires were generally lighter and younger than the resident dominant male, suggesting dominant females were unlikely to be “trading up.” Our results contrast with many socially monogamous birds, where EP sires tend to be heavier and older than the female’s current partner (Akçay and Roughgarden 2007; Cleasby and Nakagawa 2012; Hsu et al. 2015). However, this difference is likely a consequence of meerkat biology rather than a female preference for younger or lighter mates. Dominant male meerkats are usually older and heavier than other males in the population. Additionally, roving by EG males is energetically costly, resulting in weight loss (Young et al. 2005). Together, these factors explain why EG sires (who were always subordinates from other groups) tended to be younger and lighter than the resident dominant male. From the perspective of the female, dominant males would appear to be the superior mate choice, having proven their ability to survive, acquire dominance, and maintain good physical condition, making the good genes hypothesis an unlikely explanation for EPCs with “inferior” EP males.

We detected no indirect genetic benefits of EPCs, with EG-EPY and WPY not differing in terms of survival, weight, or dominance acquisition. Our results contrast with those of some other cooperatively breeding mammals. For example, in alpine marmots, EPY have greater survival than WPY in their first few years of life and a higher chance of becoming dominant (Cohas, Bonenfant, et al. 2007). Similarly, banded mongoose pups sired by EG males are heavier and more likely to survive to independence (Nichols et al. 2015). In birds, however, EPY survival advantages vary between species (Akçay and Roughgarden 2007). EPY and WPY meerkats could differ in survival before emergence or survival after 1 yr, as these are more difficult to measure due to underground rearing and adult dispersal, respectively. The stochastic nature of dominance acquisition (Hodge et al. 2008; Spong et al. 2008; Duncan et al. 2018, 2023) may also reduce the ability for heritable effects to manifest.

Female choice is commonly expected to provide considerable fitness benefits; however, as long as females can avoid close relatives and males compete for access, the benefits of mate choice might often be minimal. This may be particularly likely where the amount of care offspring receive and their growth and survival depend on group size and the number and generosity of alloparents rather than on the direct or indirect contributions of male breeding partners. The benefits of mate choice to females may consequently be smaller in cooperative breeders like meerkats than in biparental species. These females might therefore simply mate with any available male during estrus or may seek out multiple mates indiscriminately as a bet-hedging strategy. This bet-hedging approach could help guard against uncertainty in male fertility, while also increasing offspring diversity, enhancing the likelihood that at least one offspring will be well-suited to unpredictable future environmental conditions, like those found in the Kalahari.

### Individual and social influences on EPP

The ability of dominant males to guard against EG-EPP was strongly related to their energetic state. Repelling EG competitors is energetically costly in meerkats: heavier males lead chases of EG rovers more frequently, and the daily weight gain of dominant males decreases by 60% on days when EG rovers are observed at the group (Mares et al. 2012). Mate guarding may also involve dominant males scent marking (Jordan 2007; Jordan et al., 2007; Mares et al. 2011) and maintaining close constant proximity to the dominant female during estrus (Jordan et al. 2007), behaviors which could be energetically costly and detract from foraging time. Heavier dominant males, presumably possessing greater energetic stores, may be better equipped to engage in these energetically costly behaviors, which would explain why they were less likely to be cuckolded by EG males. This idea was supported in socially monogamous round-eared sengis (*Macroscelides proboscideus*), where mate guarding was shown to be energetically costly, and males who were in better body condition guarded their females more intently (Schubert et al. 2009).

The ability of dominant females to acquire EG-EPP was also associated with their energetic state, with the litters of heavier females being more likely to contain EG paternity. Females in several species appear to actively engage in seeking out EPCs through behaviors such as forays (birds: Westneat and Stewart 2003; banded mongooses: Cant et al. 2002) or remaining near territory borders to increase the chances of encountering EG males (Ethiopian wolves, *Canis simensis*: Sillero-Zubiri et al., 1996). In meerkats, EPCs and the degree to which dominant females actively seek out EG mates is not well understood, especially since much of mating occurs underground. There have, however, been some observations of dominant (and subordinate) females sneaking away from their group with EG rovers for between 1 and 24 h to copulate (Young et al. 2007). Dominant females may also increase their vigilance to detect EG males, since experiments have shown that after being presented with the scents of unfamiliar males, dominant females were more vigilant if they were likely to be seeking an EG mate (ie if they were paired with a natal dominant male) (Leclaire, Nielsen, Thavarajah, et al. 2013). Additionally, dominant female meerkats could be using scent marking to advertise to EG males, as has been suggested in other mammals such as Alaskan moose (*Alces alces gigas*) (Bowyer et al. 1994) and meadow voles (*Microtus pennsylvanicus*) (Ferkin et al. 2004), though this possibility requires further investigation. Short consortships, increased vigilance, and scent marking are all behaviors which could take time away from foraging and incur energetic costs. If some or all of these costly behaviors are involved in females seeking out or engaging in EPCs, heavier females would have a better capacity to bear these costs.

Along with individual condition, social conditions also influenced EG-EPP, with EG-EPP increasing as the availability of EG mates increased. As more roving EG males visited the group, dominant female litters were more likely to contain EG paternity, consistent with previous results from Spong et al. (2008). Our analysis could only account for identifiable rovers seen while an observer was at the group, so this effect may be even stronger when considering visits by unmarked rovers and visits that occur outside of observer presence. EP mate availability was similarly seen to be an important determinant of EPP rates in other species such as the grass wren (Cistothorus platensis) and eastern bluebird (Sialia sialis), where EPP increases with local breeding density (Stewart et al. 2009; Arrieta et al. 2022).

EPP from EG males increased when there were few subordinate males in the group to help guard against them. In meerkats, subordinate males play an important role in repelling EG males (Mares et al. 2012), likely for two main reasons: i) to prevent rovers from immigrating, as dominance takeovers by immigrant males often lead to the expulsion of resident males (Mares et al. 2012), and ii) if subordinate males are offspring of the dominant pair, they may help mate-guard their mother to ensure the production of full-siblings over half siblings (Welbergen and Quader 2006). Though dominant males were less vulnerable to EG-EPP when groups contained more subordinate males, the protective effect of subordinates plateaued, indicating that beyond a certain point, adding more subordinate males did not further strengthen defense against EG competitors. In cooperatively breeding alpine marmots, where most EPP likewise originates from EG males, EPP increased as the number of subordinate males increased, with no EPP occurring in groups with no subordinate males (Cohas et al. 2006). This difference from our results is likely explained by subordinate male alpine marmots playing little role in group defense against conspecifics (Arnold 1990), unlike in meerkats. Although subordinate male meerkats can protect dominant males against EG cuckoldry, they can also act as reproductive competition themselves, siring 6.9% of the dominant female’s pups in groups where at least one subordinate immigrant male was present. Consequently, greater numbers of subordinates could expose dominant males to a different type of paternity threat, though we did not directly statistically test this due to small sample sizes for IG-EPP.

## Conclusion

EPP does not appear to provide indirect genetic benefits to dominant female meerkats in the form of “good genes” or “compatible genes” when the female has an unfamiliar dominant male partner available. Instead, intrapopulation variation in EPP was best explained by EP mate availability, the ability of dominant males to repel these competitors, and the energetic capacity for females to find and/or engage in these EPCs. Our results help explain the pattern of EPP in meerkats but do not identify the evolutionary benefits females gain from EPP when paired with an unfamiliar dominant male. Comparative analyses of socially monogamous species have also failed to find consistent support for the good genes or compatible genes hypotheses. Females may not be gaining any benefits from EPP or could be using EPP as a bet-hedging strategy to protect against uncertainty in their environment or their paired mate, and we suggest future EPP research targets bet-hedging for further examination. Our study shows the importance of considering a wide range of genetic, social, and individual-based factors to understand the patterns of EPP within a population and the evolutionary drivers of this EP mating behavior.

## Supplementary Material

araf016_suppl_Supplementary_Materials

## Data Availability

Analyses reported in this article can be reproduced using the data provided by Herdtle et al. (2025).
