# Supplementary material for: Patterns and drivers of female extra-pair mating in wild Kalahari meerkats
Source: Behav Ecol. 2025 Mar 6;36(3):araf016. doi: 10.1093/beheco/araf016 (PMC11959362; doi:10.1093/beheco/araf016)
Supplement: araf016_suppl_Supplementary_Materials [file araf016_suppl_supplementary_materials.docx]

**CERVUS exclusion analysis simulation parameters**

Parameters for the simulation of paternity analysis were chosen following procedures by Lemons et al. (2015). The number of candidate fathers was set to 100, which was the mean number of known candidate fathers for each pup in the COLONY2 analyses (Nielsen et al. 2012). The proportion of loci typed was 0.9499, and the proportion of loci mistyped was 0.0343, an average calculated from the locus-based genotyping error rates in Nielsen et al. (2012). The minimum number of typed loci was set to 9 (half of the total 18 loci). In the first simulation iteration, the proportion of candidate parents sampled was set to 0.95, which is the complement to Spong et al.’s (2008) estimate that 5% of dominant females’ offspring are sired by EG males (Lemons et al. 2015). To ensure this initial estimate did not influence the final outcome, we repeated the simulation and paternity analysis process three times – each time we used the results of the current iteration to calculate the overall EG-EPP rate in our dataset and then used this estimate in the next iteration’s simulation (Lemons et al. 2015). Reported results are based on the third and final iteration.

**Table S1: EG-EPP Model Output**

| **Predictor** | **Estimate** | **Est. Error** | **95% CI Lower** | **95% CI Upper** |
| --- | --- | --- | --- | --- |
| Intercept | -5.99 | 2.07 | -10.49 | -3.00 |
| Dominant female natal status: non-natal | -3.35 | 2.19 | -8.24 | 0.37 |
| Pair relatedness | -1.22 | 1.92 | -5.36 | 2.22 |
| **Dominant male weight** | **-3.80** | **1.90** | **-8.21** | **-0.62** |
| **Dominant female weight** | **3.21** | **1.64** | **0.35** | **6.81** |
| **EG rovers** | **1.61** | **0.84** | **0.06** | **3.41** |
| Adult subordinate males | -1.56 | 1.48 | -4.72 | 1.28 |
| **(Adult subordinate males) ^2^** | **2.96** | **1.39** | **0.25** | **5.86** |
| Dominant male tenure length | -0.75 | 1.32 | -3.50 | 1.76 |

Results from the Bayesian EG-EPP model predicting the likelihood of a litter containing EPP from an EG male (n = 229 litters). The table presents the mean estimate, estimated error, and the lower and upper bounds of the 95% credible intervals for each fixed effect. The model used a Bernoulli distribution and included random effects for dominant female identity and dominant pair identity. All continuous predictor variables were centered and scaled by 2 SD prior to modeling. Estimates are reported on the logit link scale, representing a change in the log-odds of EG paternity in response to a 2SD increase in the continuous predictor or a change in ‘dominant female natal status’ from the reference category of ‘natal’ to the category ‘non-natal’. Coefficient estimates whose 95% credible intervals do not cross zero are bolded.



**Figure S1.** Post-hoc examination of the non-linear relationship between the number of adult subordinate males and the likelihood of EG paternity in a litter. a) Results from the ‘fewer males’ model, incorporating groups with less than 5.3 subordinate males at conception (n = 171 litters). b) Results from the ‘many males’ model, incorporating groups with more than 5.3 subordinate males at conception (n = 58 litters). The plots depict predicted mean probability estimates (lines) and 95% credible intervals (shaded regions), with vertical ticks representing individual litters. Predictions were calculated while holding all other continuous predictors constant at their means and setting dominant female natal status to natal.

**Conservative relatedness analyses**

When conservatively restricting the EG-EPP analysis to only instances where both dominants had two parents with known identities and, therefore, a more certain pedigree, pair relatedness did not have any clear directional effect on the likelihood of EG-EPP (Tab. S2), consistent with results from the non-conservative model (Tab. S1). The reduced sample size for this conservative model (from 229 to 101 litters) meant that it struggled to converge when all the predictors were included; therefore, these results are from a simplified model with no quadratic terms, where the only fixed effects besides relatedness were predictors that showed a clear directional effect on EG-EPP in the original model.

When only examining the six EG-EPP instances where the dominant male, dominant female, and EG sire each had two parents with known identities, EG sires did not differ significantly from the resident dominant males in terms of pedigree relatedness to the dominant female (Wilcoxon signed-rank: W = 5, *P* = 0.423; EG sire mean = 0.201, dominant male mean = 0.243, mean paired difference = 0.042), consistent with results from the non-conservative comparison.

**Table S2: Conservative EG-EPP Model Output**

| **Predictor** | **Estimate** | **Est. Error** | **95% CI Lower** | **95% CI Upper** |
| --- | --- | --- | --- | --- |
| Intercept | -23.18 | 21.84 | -88.57 | -5.55 |
| Pair relatedness | 5.43 | 12.62 | -14.03 | 39.50 |
| **Dominant male weight** | **-16.83** | **17.20** | **-64.25** | **-2.18** |
| Dominant female weight | 15.00 | 17.55 | -0.50 | 62.72 |
| EG Rovers | 0.32 | 6.74 | -12.68 | 12.22 |

Results from the conservative Bayesian EG-EPP model predicting the likelihood of a litter containing EPP from an EG male after restricting the dataset to pairs where both dominants had two identified parents (n = 101 litters). The table presents the mean estimate, estimated error, and the lower and upper bounds of the 95% credible intervals for each fixed effect. The model used a Bernoulli distribution and included random effects for dominant female identity and dominant pair identity. All continuous predictor variables were centered and scaled by 2 SD prior to modeling. Estimates are reported on the logit link scale, representing a change in the log-odds of EG paternity in response to a 2SD increase in the continuous predictor. Coefficient estimates whose 95% credible intervals do not cross zero are bolded.

**Table S3: Model outputs comparing EG-EPY and WPY maternal half-siblings**

|  | **Predictor** | **Estimate** | **Est. Error** | **95% CI Lower** | **95% CI Upper** |
| --- | --- | --- | --- | --- | --- |
| **a)** | **Survival from Emergence to 90 days** (n = 250 pups) | |  |  |  |
|  | Intercept | 2.53 | 0.86 | 0.93 | 4.38 |
|  | Father type: EG | 0.35 | 1.15 | -1.81 | 2.64 |
|  | **Number of pups** | **-2.31** | **0.85** | **-4.08** | **-0.79** |
|  | Number of helpers | -0.20 | 1.07 | -2.41 | 1.74 |
| **b)** | **Survival from 90 to 365 days** (n = 217 pups) |  |  |  |  |
|  | Intercept | 2.81 | 0.56 | 1.79 | 4.03 |
|  | Father type: EG | -0.17 | 0.82 | -1.68 | 1.59 |
|  | **Offspring sex: male** | **-0.94** | **0.46** | **-1.87** | **-0.06** |
|  | **Number of pups** | **1.99** | **0.48** | **1.09** | **2.98** |
|  | Number of helpers | 0.81 | 0.56 | -0.24 | 2.00 |
| **c)** | **Dominance Acquisition** (n = 166 pups) |  |  |  |  |
|  | Intercept | -1.36 | 0.36 | -2.09 | -0.66 |
|  | Father type: EG | -0.05 | 0.76 | -1.68 | 1.32 |
|  | Offspring sex: male | -0.63 | 0.46 | -1.58 | 0.22 |
| **d)** | **Weight at 90 days** (n = 189 pups) |  |  |  |  |
|  | Intercept | 328.74 | 10.28 | 308.10 | 348.44 |
|  | Father type: EG | 4.35 | 11.52 | -18.11 | 26.86 |
|  | Offspring sex: male | 2.54 | 5.68 | -8.45 | 13.53 |
|  | Mean age at weights | 6.52 | 6.84 | -6.94 | 20.19 |
|  | **Number of pups** | **-23.02** | **7.99** | **-38.76** | **-7.74** |
|  | **Number of helpers** | **-24.15** | **10.30** | **-45.07** | **-5.00** |
|  | **Rainfall** | **42.39** | **14.34** | **17.59** | **73.84** |
|  | **(Rainfall) ^2^** | **-30.66** | **14.67** | **-61.41** | **-3.10** |
| **e)** | **Weight at 365 days** (n = 154 pups) |  |  |  |  |
|  | Intercept | 588.70 | 13.37 | 562.59 | 615.47 |
|  | Father type: EG | -24.09 | 18.55 | -59.45 | 12.50 |
|  | **Offspring sex: male** | **20.95** | **9.75** | **2.08** | **39.60** |
|  | Mean age at weights | 6.28 | 10.79 | -14.63 | 27.08 |
|  | Number of pups | 12.07 | 13.08 | -13.28 | 38.77 |
|  | **Number of helpers** | **-30.50** | **14.82** | **-59.18** | **-1.29** |
|  | **(Number of helpers) ^2^** | **-36.27** | **18.44** | **-71.87** | **0.42** |
|  | Rainfall | 12.73 | 16.04 | -19.31 | 44.84 |

Results from Bayesian mixed-effects models fitted using Bernoulli (logit link) (a, b, c) or Gaussian (identity link) (d, e) distributions. The table presents the mean estimate, estimated error, and the lower and upper bounds of the 95% credible intervals for each fixed effect. All models included dominant pair identity as a random effect, while models a, b, d, and e also included breeding year as a random effect and birth month as a cyclic cubic spline. All continuous predictor variables were centered and scaled by 2 SD prior to modeling. Coefficient estimates for ‘EG’ father type and ‘male’ sex are shown, with ‘dominant’ father type and ’female’ sex as reference categories. Estimates represent a change in the log-odds of survival (a, b), log-odds of dominance acquisition (c), or weight (in grams) (d, e) in response to a 2SD increase in the continuous predictor, or a change from the reference category to the specified category for categorical predictors. Coefficient estimates whose 95% credible intervals do not cross zero are bolded.
